# A novel strategy and kinetics analysis of half‐fractional high cell density fed‐batch cultivation of *Zygosaccharomyces rouxii*

**DOI:** 10.1002/fsn3.666

**Published:** 2018-04-30

**Authors:** Zhijiang Li, Yanan Zhou, Hongzhi Yang, Dongjie Zhang, Chengtao Wang, Hong Liu, Xin Li, Jing Zhao, Chunhong Wei

**Affiliations:** ^1^ Department of Food and Engineering College of Food Heilongjiang Bayi Agricultural University Daqing China; ^2^ Beijing Engineering and Technology Research Center of Food Additives Beijing Technology& Business University (BTBU) Beijing China

**Keywords:** half‐fractional fed‐batch, high cell density culture, kinetics, *Zygosaccharomyces rouxii*

## Abstract

*Zygosaccharomyces rouxii* is an important microorganism for aroma production in traditional fermented foods. Using *Z. rouxii* as the original strain, the batch was split after glucose depletion in the culture medium. Half of the volume of the culture medium was released, and fresh culture medium was fed in. The exponential culture kinetics and the formula for the half‐fractional fed‐batch cultivations were determined to achieve a new strategy for high cell density culturing of *Z. rouxii*. Based on a full cultivation, three half‐fractional fed‐batch cultivations were performed after every 10 hr of culture. The specific growth rates of *Z. rouxii* at the different stages were in the order μ_*X*0_>μ_*X*1_>μ_*X*2_>μ_*X*3_ (0.525 to 0.229 hr^−1^). The glucose substrate consumption rates gradually decreased following the order μ_*S*0_>μ_*S*1_>μ_*S*2_>μ_*S*3_ (−1.165 to −0.722, g/g). The equation models for cell growth and glucose substrate consumption showed typical exponential behavior. The total cell yield was 1.78‐fold higher than the yield from a full cultivation, and this continuous subculture strategy also indicated a higher efficiency than traditional full cultivation. A new strategy for highly efficient culturing of *Z. rouxii* was achieved in a pilot scale. A foundation with data support for the production and application of *Z. rouxii* was developed.

## 1 INTRODUCTION

*Zygosaccharomyces rouxii* is a common osmotolerant yeast. During the fermentation of soybean paste and soy sauce, *Z. rouxii* generates furanone compounds that give a caramel‐like smell, making *Z. rouxii* an important aroma‐producing microbe (Dakal, Solieri, & Giudici, 2014; Ohata, Kohama, Morimitsu, Kubota, & Sugawara, 2007). Therefore, exploring large‐batch high cell density cultivation (HCDC) of *Z. rouxii* could provide effective technical support for the preparation and application of direct vat inoculation. The results could be beneficial for industrial use, improve subculture efficiency, and achieve aroma enhancement.

High cell density cultivation is one focused research topic in the international bioengineering field. The purpose was to produce the end product in as great an amount or with as high an efficiency as possible by increasing the cell density (Riesenberg & Guthke, 1999). In 1973, Japanese scholar Yoshida established the first theoretical mathematical model based on fed‐batch fermentation, and studies of the kinetics advanced to the theory development stage (Yoshida, Yamane, & Nakamoto, 2010). Since that time, models and kinetic studies of various fed‐batch fermentation approaches, including continuous fermentation and batch fermentation, have been tested and applied (Shin & Lim, 2006). HCDC of yeast utilizing the fed‐batch method has provided a theoretical model and application basis for further applications (Li, Zhao, & Bai, 2007; Li et al., 2016; Miszczak, Cibis, & Krzywonos, 2012). Fed‐batch fermentation eliminates substrate inhibition, product feedback inhibition, and repression via the decomposition of metabolites. Therefore, the target strain can be obtained periodically in large quantities. Additionally, inoculation processes and contamination can be minimized with the fixed supply of fresh medium, thereby allowing the method to be employed in industrial production and applications. Li et al. (2016) employed a logistic model and logarithmic phase to perform the splits; these methods are common methods for model development for yeast fed‐batch and kinetic studies (Ângelo, Vitolo, & Pessoa, 2007; Li et al., 2016). However, these studies mainly focused on theory and small sample size testing, which could not satisfy the practical needs for industrial production.

This study was performed using a 20‐L automatic fermenter for HCDC of *Z. rouxii*. Half‐fractional fed‐batch fermentation was implemented (i.e., when the medium nutrients were depleted, half of the volume of the yeast culture was removed, and fresh medium of equal volume was added to the fermenter to ensure sufficient nutrients and continual fermentation). From the standpoint of industrial production, half‐fractional fed‐batch HCDC is easy to implement, requires a low equipment investment, and results in a higher production efficiency. The goal of obtaining multiple high biomass gains for *Z. rouxii* with one inoculation can be achieved. Furthermore, the *Z. rouxii* growth rate and the substrate consumption kinetic model of the logistic model‐based half‐fractional fed‐batch cultivation were explored. This study provides a theoretical basis for the cultivation method and technical support for batch production of *Z. rouxii* and its applications.

## 2 MATERIALS AND METHODS

### 2.1 Strain and medium

*Zygosaccharomyces rouxii* (product no. 32899) was obtained from the China Center of Industrial Culture Collection. Yeast extract–peptone–dextrose (YPD) broth (20.0 g/L peptone, 20.0 g/L glucose, and 10.0 g/L yeast extract) was obtained from Qing Dao Hope Bio‐Technology Co., Ltd.

### 2.2 *Z. rouxii* culture expansion

*Zygosaccharomyces rouxii* freeze‐dried powder was activated. A sample was added to 100 ml of sterilized YPD broth in a bioclean room and placed into a temperature‐controlled shaking incubator for 3 days at 28°C and 180 rpm. The total cell count was monitored, and the culture was reserved for later use when the cell count reached 10^8^ CFU/ml.

A total of 100 ml of seed medium was added to a 250‐ml flask and inoculated with 5% activated seed. The flask was cultured for 30 hr at 28°C under 180 rpm in a temperature‐controlled shaking incubator.

### 2.3 *Z. rouxii* culture growth and half‐fractional fed‐batch method

For a full cultivation, the inoculation size was set as 5% in 20‐L fermenter and cultured at 28°C, 500 rpm, pH 5, and a DO value of 30%; additionally, 200 ml of soybean oil was added to fermenter as a defoaming agent. The culture was sampled every 3 hr to analyze the glucose content in the medium and to determine the cell dry weight. The fermentation period was 30 hr.

**Figure 1:**
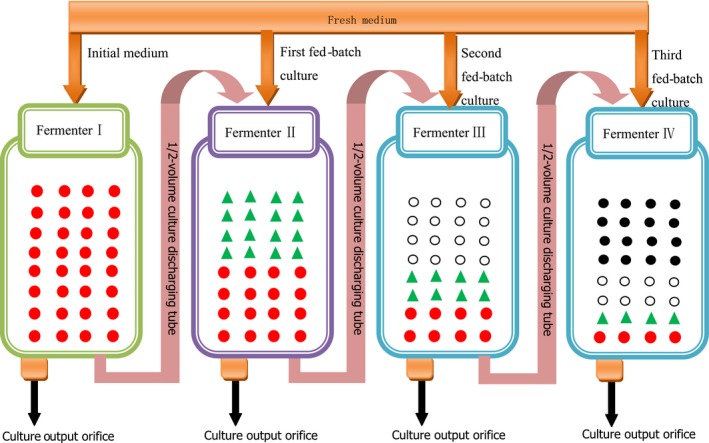
Schematic diagram for the half‐fractional fed‐batch HCDC of *Zygosaccharomyces rouxii*. 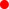, denotes the initial medium; 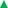, denotes the first fed‐batch fresh medium; 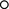, denotes the second fed‐batch fresh medium; 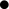, denotes the third fed‐batch fresh medium

For the half‐fractional fed‐batch cultivation, the inoculation condition was same to the full cultivation except that the culture was sampled every 2 hr. When the glucose content was depleted to 10 hr and at the level of below 2 g/L, half of the fermentation broth volume was removed from the fermenter, and an equal volume of fresh medium was added. This process was repeated three times. Based on the economic efficiency of the industry, the continuous fermentation period was set at 40 hr including 10 hr of stage 0 and three 10 hr equal of stages 1–3. The specific operation procedure is shown in Figure 1.

### 2.4 Measurement of glucose in the medium and the cell dry weight

The glucose content was determined with a colorimetric method using Somogyi–Nelson method (Hatanaka & Kobara, 2008). Briefly, 0.5 ml of the culture and equal Somogyi's regent was mixed to perform for 20 min in boiling water and then cooling in cold water. 2 ml of Folin–Ciocalteu phenol reagent was added and stirred. After adding 5 ml of water, absorbance was determined at 660 nm in a 1‐cm cell with spectrophotometer and calculated the content of glucose with standard curve.

A 5‐ml aliquot of the *Z. rouxii* fermentation culture was placed into a dried and weighed centrifuge tube and centrifuged for 10 min at 7,168 g. The *Z. rouxii* sample was collected, washed twice with deionized water, centrifuged, dried in a drying oven at 80°C, and weighed; then, the cell dry weight was calculated.

### 2.5 Calculations for the half‐fractional fed‐batch cultivation

#### 2.5.1 Cell growth kinetics equations

The dry weight gained in each batch of *Z. rouxii* culture was used to determine the specific growth rate μ_*X*_ (unit: h^−1^). The equation is as follows:

(1) $$\mu_{X}\;=\;\left(\frac{1}{X}\right)\left(\frac{\mathrm{dX}}{\mathrm{dt}}\right)\;=\;\frac{1}{X}\frac{\Delta X}{\Delta t}$$

where *X* is the biomass (g/L) and *t* is the time.

In the experiments, substrate inhibition was eliminated because feeding was performed according to an exponential growth strategy (i.e., the cultures were fed when the culture medium was depleted). Therefore, cell growth can be calculated using the following formula (Amin, 2014; Li et al., 2016):

$$\frac{d(XV)}{\mathrm{dt}}\;=\;\mu_{X}\left(XV\right)$$

$\frac{d(XV)}{\mathrm{XV}}\;=\;\mu_{X}dt$; by integrating both sides of the equation, we obtain the following equation:

$$XV\;=\;XV_{0}e^{\mu_{X}\left(t-t_{0}\right)}$$

Due to the use of half‐fractional feeding, the volume did not change after each addition of medium. The formula can be re‐arranged to $X=X_{0}e^{\mu_{X}\left(t-t_{0}\right)}$, where *t*_0_ is the initial time and is set as zero (*t*_0_ = 0). Thus, the growth kinetic formula for *Z. rouxii* can be expressed as

(2) $$X\;=\;X_{0}e^{\mu_{X}t}$$

#### 2.5.2 Kinetics formula of substrate consumption

The following formulas were employed for the calculation of substrate consumption (Amin, Alotaibi, Youssef, & Saleh, 2008):

$$S_{t}\;=\;\frac{V_{0}X_{0}\left(e^{\mu_{S}t}-1\right)}{Y_{X/S}}\;+\;S_{0}$$

(3) $$S_{t}\;=\;\frac{20X_{0}\left(e^{\mu_{S}t}-1\right)}{Y_{X/S}}+S_{0}$$

Biomass yield coefficient calculation:

(4) $$Y_{X/S}=\frac{\Delta \text{Dry}\;\text{Cell}\;\text{Weight}}{\Delta \text{Glucose}}$$

where μ_*S*_ is the substrate consumption rate (the ability to consume substrate per unit time, h^−1^) and is calculated using ln *S *= μ_*S*_ *t* + *a* (*a* is the intercept, and μ_*S*_ is the slope); *S*_*t*_ and *S*_0_ are the substrate glucose contents (g) at times *t* and *t*_0_, respectively; *V*_0_ and *X*_0_ are the volumes (20 L) at *t*_0_ and the biomass concentration (g/L) at *t*_0_, respectively; and *Y*_X/S_ is the biomass yield coefficient (g/g).

### 2.6 Data processing

All parameters were measured three times and averaged. Figures and data processing were performed using OriginPro 9 software.

## 3 RESULTS AND DISCUSSION

### 3.1 Analysis of *Z. rouxii* culture growth kinetics

**Figure 2:**
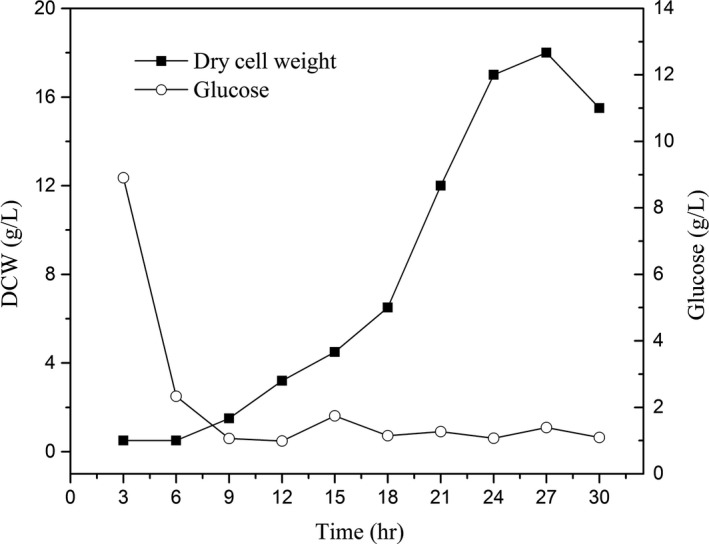
Changes in the glucose substrate content and cell dry weight of *Zygosaccharomyces rouxii* in a full cultivation

*Zygosaccharomyces rouxii* was cultured in a 20‐L fermenter. The changes in the glucose content and the cell dry weight during a full cultivation process are illustrated in Figure 2. The cultivation of *Z. rouxii* showed a typical “S‐shape” growth curve. The cell dry weight–time curve and medium glucose content–time curve were correlated during the cell growth period. Those results were similar to the reports and could be used for the following study (Jiang et al., 2013; Li et al., 2016). *Z. rouxii* was in the lag phase at the initial fermentation stage; thus, the changes in the cell dry weight and glucose content were slow. At 6 hr of cultivation, the medium glucose content started to decrease rapidly; at this point, cell production increased rapidly and entered the exponential phase. At 24–27 hr of cultivation, the medium glucose was depleted below the level of 2 g/L, and the biomass, cell dry weight, and metabolic products of *Z. rouxii* were at their maximum levels; at this point, the process was in the rapid growth phase and the main fermentation stage. During the late stage of fermentation, the accumulation of products inhibited cell growth; thus, the cell dry weight showed a decreasing trend. From the process of *Z. rouxii* cultivation, we observed that there was a rapid decrease in nutrients inside the fermenter before exponential growth and that glucose was depleted at 10 hr of cultivation below the level of 2 g/L. To obtain a higher biomass and perform half‐fractional fed‐batch cultivation, a culture split was performed at 10 hr.

### 3.2 Analysis of *Z. rouxii* cell growth

**Figure 3:**
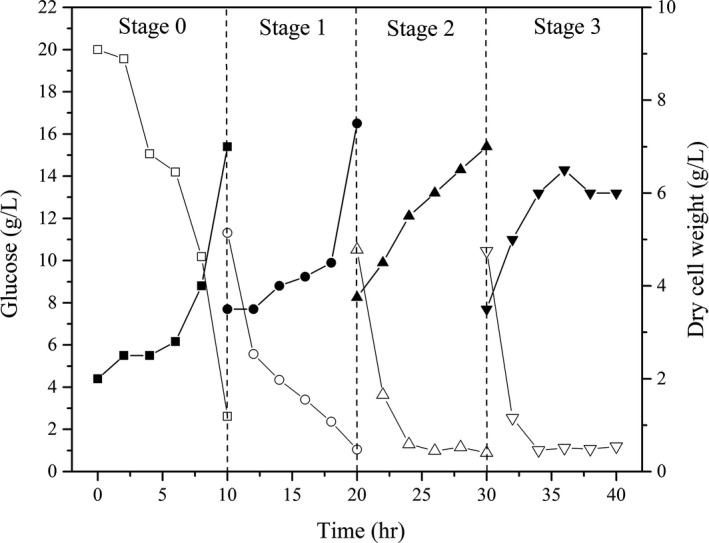
Plots for half‐fractional HCDC of *Zygosaccharomyces rouxii*. Stage 0, Stage 1, Stage 2 and Stage 3 represent the initial cultivation and the 1st to 3rd half‐fractional feeds, respectively. Open markers represent the glucose changes in each stage, and solid markers represent the changes in the cell dry weights of *Z. rouxii*

Based on the change in the glucose substrate content during *Z. rouxii* cultivation, culture splits were performed after every 10 hr of cultivation for each batch for a total of 3 splits. The initial cultivation and each split cultivation are denoted as Stage 0, Stage 1, Stage 2, and Stage 3. The results are shown in Figure 3. At each feed, the *Z. rouxii* biomass, cell dry weight, and medium concentration were half of the initial value.

At Stage 1, half of the culture volume was removed with the addition of fresh medium in the same volume; thus, at 12 hr of cultivation, the glucose level increased, and the cell dry weight decreased. As the fermentation time increased (at 14–18 hr), the medium glucose content showed a decreasing trend; conversely, the cell dry weight was first steady and then increased. Substrate inhibition was eliminated by the addition of fresh medium in half of the volume, which was beneficial for *Z. rouxii* biomass accumulation. Meantime cell adaptations for metabolic and environmental stress were eliminated, but not completely inhibited (Burgard, Valli, Graf, Gasser, & Mattanovich, 2017). This supplement strategy could be more useful to reduce the nutrient limitation and produce more biomass and production for *Z. rouxii*.

At Stage 2, the cell dry weight increased rapidly, and the glucose concentration decrease rate was obviously higher than in Stage 1. Stages 2 and 3 showed the same decreasing trend in glucose content. Simultaneously, the glucose contents were depleted to below to 2 g/L at stages 1–3 and similar to those in full cultivation (Figure 2). However, after 36 hr, the cell dry weight no longer increased but instead decreased. This trend could be continuously accumulating results of the nutrient limitation (Burgard et al., 2017) or production inhibition of metabolites such as ethanol or others (Al‐Tabib, Al‐Shorgani, Hasan, Hamid, & Kalil, 2017). Although the physical and biochemical environments of the fed‐batch cultivation differ from those of a full cultivation, it is a useful attempt to build *Z. rouxii* high‐density cultivation.

**Figure 4:**
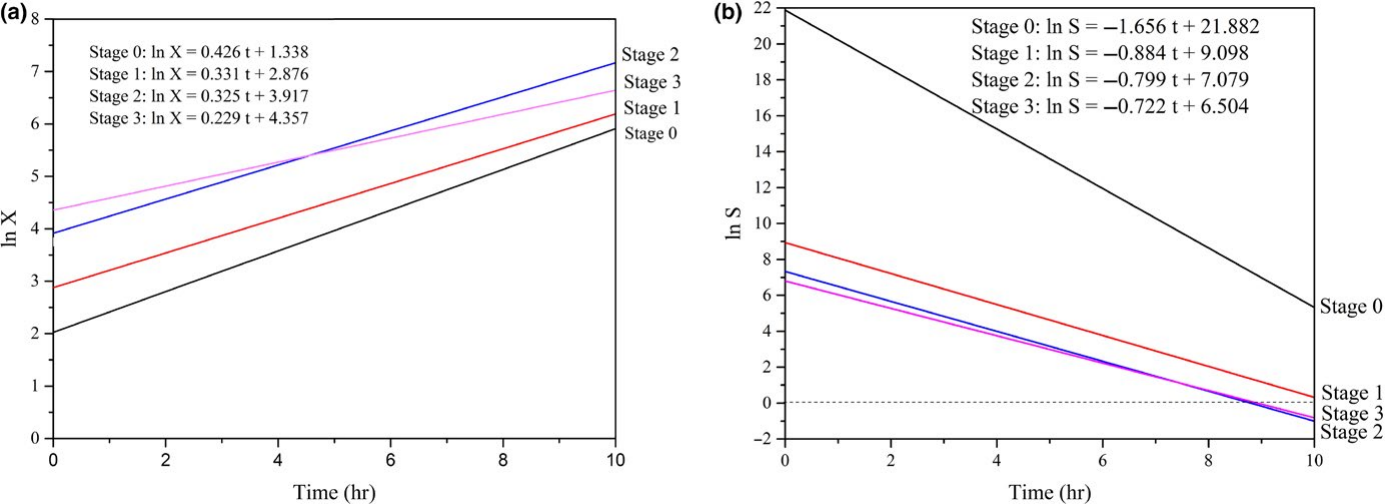
Kinetics plot of half‐fractional fed‐batch HCDC of *Zygosaccharomyces rouxii* (a) cell growth, (b) substrate consumption. HCDC, high cell density cultivation

As seen in Table 1, the specific growth rates of *Z. rouxii* at various stages trended as follows: μ_*X*0_ > μ_*X*1_ > μ_*X*2_ > μ_*X*3_ (0.525 to 0.229, h^−1^). This result indicated that the cell mass at each hour gradually increased along with each feed, whereas the cell growth rate showed a gradually decreasing trend. After each addition of fresh medium, the glucose concentration rapidly returned to the concentration at the last feed. The nutrients required by the cells rapidly returned to the initial status. A new continuous fed‐batch cultivation with starters from the former stage and fresh culture was rebuilt with the low contaminant unlike discontinuous method (Sung, Lee, Kim, Nam, & Chang, 2017). At this point, the cell concentration or the amount of the inoculum reached 0.606 × 10^8^, 0.775 × 10^8^, and 1.320 × 10^8^ cells/ml. As shown in Figure 4a, when *t* = 0, the intercepts of ln X gradually increased (1.338–4.357) at stages 0, 1, 2, and 3; this increase in the intercept provided further support for the results mentioned earlier. Therefore, the increasing amount of the inoculum resulted in relatively insufficient nutrient contents and influenced the increase in the cell growth and cell numbers compared with the initial steady medium concentration. The half‐fractional fed‐batch strategy is different from other reported methods. Li et al. (2016) studied the fix‐specific growth rate of *Z. rouxii* (μ = 0.05 hr^−1^) using an exponential feeding strategy; after 42 hr of fermentation, the cell dry weight obtained was as high as 72.96 g/L, which was higher than the cell dry weight obtained in the current study. During half‐fractional fed‐batch cultivation, *Z. rouxii* was cultured to the exponential stage; then, the half‐fraction of the medium and cells was released when entering the steady stage. The process did not go through a steady stage, and the culture volume was fixed. The differences in the cultivation time, cultivation stage, and feeding strategy resulted in differences in the cell dry weights (Kim, Lee, Lee, Chang, & Chang, 2004; Kishimoto, Omasa, & Katakura, 2005).

**Table 1:** *Zygosaccharomyces rouxii* cell growth with half‐fractional HCDC and substrate calculation

	Stage 0	Stage 1	Stage 2	Stage 3	One full cultivation
Initial substrate concentration *S*_0_ (g/L)	20.00	11.311	10.525	10.444	20.00
Ending substrate concentration *S*_*t*_ (g/L)	2.621	1.050	0.887	1.193	0.642
Initial cell dry weight *X*_0_ (g/L)	2.008	3.505	3.750	3.510	2.008
Ending cell dry weight *X*_*t*_ (g/L)	7.010	7.500	7.020	6.001	15.500
Initial cell count (×10^8^ cells/ml)	0.100	0.606	0.775	1.320	0.100
Ending cell count (×10^8^ cells/ml)	1.211	1.540	2.640	2.450	2.670
Specific cell growth rate μ_*X*_ (h^−1^)	0.525	0.331	0.325	0.229	—
Cell growth kinetics equation	X = e^0.525*t*^	X = e^0.331*t*^	X = e^0.325*t*^	X = e^0.229*t*^	—
Substrate consumption rate μ_*s*_ (h^−1^)	−1.656	−0.884	−0.799	−0.722	—
Product yield coefficient *Y*_*X*/*S*_ (g/g)	−0.2878	−0.389	−0.339	−0.269	—
Substrate consumption kinetics equation	*S*_t_ = −139.54e^−1.656^ + 159.540	*S*_t_ = −80.20e^−0.884*t*^ + 191.517	*S*_t_ = −221.24e^−0.799*t*^ + 231.764	*S*_t_ = −260.97e^−0.722*t*^ + 271.411	—

Half‐fractional fed‐batch cultivation of *Z. rouxii* allowed the elimination of the intense inoculation steps during the culture processes and reduced problems with contamination from infectious microbes, which was beneficial for the application in a pilot scale. As seen in Table 1, the cell growth kinetics of *Z. rouxii* showed a typical exponential trend with the adaptation of the exponential feed.

### 3.3 Analysis of glucose substrate consumption

As shown in Table 1, the substrate consumption rate at various stages followed the trend μ_*S*0_ > μ_*S*1_ > μ_*S*2_ > μ_*S*3_ (−1.165 to −0.722, g/g). This trend suggests that when glucose is consumed in the unit substrate, the *Z. rouxii* cell number increase rate slows as the feed is performed. This result is consistent with the decrease in the cell growth rate mentioned above. The product yield coefficients showed the trend Y_*X*/*S*1_ > Y_*X*/*S*2_ > Y_*X*/*S*3_ (−0.389 to −0.269). This result suggests that the substrate consumption gradually decreased in stages 1 to 3 when the same amount of *Z. rouxii* cells were obtained. As illustrated in Figure 4b, when *t* = 0, the intercepts of ln *S* in stages 0, 1, 2, and 3 also gradually decreased (21.882 to 6.504). At Stage 0, the amount of the inoculum was lower but the glucose concentration was twice the concentration in the other stages, leading to a lower product yield coefficient (*Y*_*X*/*S*0_) (Table 1). Therefore, the kinetics analysis for this half fed‐batch presents a theoretical base for further application (Sansonetti, Hobley, Calabrò, Villadsen, & Sin, 2011).

In accordance with the cell growth, the consumption of glucose substrate at the different time points and stages also showed a typical negative exponential trend (Table 1). Similar to other reports, glucose was consumed at an exponential rate for cell growth and division when entering the steady phase from the lag phase. The *Z. rouxii* cell numbers started to accumulate, and metabolic products were formed (Cheng, Wu, & Chen, 2002; Moon et al., 2003).

### 3.4 Analysis of the production efficiency of *Z. rouxii* HCDC

Using a full cultivation as the reference (Table 2), the total time for stages 1–3 in the half‐fractional fed‐batch cultivation was 30 hr. The total medium used in the stages was 1.5‐fold the total medium used for a full cultivation. The culture volume was 20 L for both methods. The total cell numbers produced from all stages were 1.32‐fold the total cell numbers in a full cultivation. The *Z. rouxii* cell production contribution rates at stages 1–3 were 75.00%, 70.20%, and 60.01%, respectively, which showed a decreasing trend. This trend suggested that the cell production efficiency gradually decreased after each split and feed. The total cell amount in the four stages was 1.78‐fold the total amount in a full cultivation. And this half‐fractional fed‐batch method reduced the chance of contamination during each subculture, in contrast, full cultivation method inoculated from the fresh medium each time and indicated a discontinuous characteristic (Li et al., 2007). Therefore, this novel strategy could improve the production efficiency for *Z. rouxii*.

**Table 2:** Cell growth and substrate calculation for half‐fractional fed‐batch high cell density cultivation of *Zygosaccharomyces rouxii*

	Stage 0	Stage 1	Stage 2	Stage 3	Full cultivation
Medium volume	1.0	0.5	0.5	0.5	1.0
Cultivation time (h)	10	10	10	10	30
Culture volume (L)	20	20	20	20	20
Substrate consumption ratio (%)	86.90	90.72	91.57	88.58	96.79
Cell production contribution (%)	70.10	75.00	70.20	60.01	100
Total cells produced (g/L)	7.010	20.521			15.500

Cell production contribution = cell dry weight at the stage/cell dry weight from one full cultivation × 100%, the cell dry weight in a full cultivation was considered 100%. Substrate consumption rate = (initial − ending glucose content at a certain stage)/(initial glucose content at the same stage) × 100% (Table 1 for the specific values).

After a full cultivation, the *Z. rouxii* cells reached the steady phase and decline phase, and the substrate consumption ratio reached 96.79%. Stage 0 essentially represents a feed performed at 10 hr of full cultivation. Cell growth started to enter the steady phase when the feed was completed; hence, the substrate consumption ratio (86.90%) was lower. Three feeds were performed when the cell growth reached the exponential and steady phases. With fresh medium accounting for half of the volume and a higher initial cell count, the substrate consumption rates in stages 1 and 2 increased rapidly to 90.72% and 91.57%, respectively. In Stage 3, the increment in the cell numbers slowed down, leading to a lower substrate consumption ratio of 88.58%. After three feeds, the substrate consumption gradually decreased as the efficiency of the half‐fractional fed‐batch HCDC decreased. Therefore, the splitting method in this fed‐batch strategy is set.

## 4 CONCLUSIONS

The purpose of high‐density cultivation of microbes in industrial production is to maximize the biomass gained. Fed‐batch cultivation is a commonly used HCDC method. The microorganism production efficiency and cell dry weight are improved to eliminate substrate inhibition via the addition of fresh medium (Riesenberg & Guthke, 1999; Yamanè & Shimizu, 2006). This study targeted the HCDC of *Z. rouxii* where a culture system in steady phase was half‐fractioned, and each fraction was supplied with three feeds of fresh medium. With this method, a highly efficient cultivation with three continual batches was obtained, which formed a new strategy for the exponential cultivation of *Z. rouxii*. This development provides fundamental data and a model for the production and application of *Z. rouxii*.

The half‐fraction fed‐batch strategy is beneficial for industrial production operations. The total cell production of *Z. rouxii* was 1.78‐fold the total cell production of a full cultivation, and the production efficiency was improved. Furthermore, the split was performed during the exponential phase when *Z. rouxii* was undergoing vigorous growth; thus, the quantity of the cells and the division of the cells were secured. Cell growth was in a good condition that gave a superior strain of highly active *Z. rouxii*. Compared with the continuous and traditional batch cultivation methods, half‐fractional fed‐batch cultivation reduced the Crabtree effect, improved the production of biomass (Reynders, Rawlings, & Harrison, 1997; Shene, Mir, Andrews, & Asenjo, 2000), and was advantageous for HCDC. Due to the gradual decrease in the cell growth rate, substrate consumption, and increment in cell numbers after each split, we recommend feeding the culture three times to accomplish half‐fractional fed‐batch cultivation. Among stages 0, 1, 2, and 3, the substrate consumption ratio and cell production contribution were lowest in Stage 3. Therefore, further evidences should present to this strategy, such as *Z. rouxii* cell proliferation and metabolite ability, in which it can assess the comprehensive effects on the larger‐scale production and applications.

## ETHICAL STATEMENT

I certify that this manuscript is original and has not been published and will not be submitted elsewhere for publication while being considered by Food Science & Nutrition. And the study is not split up into several parts to increase the quantity of submissions and submitted to various journals or to one journal over time. No data have been fabricated or manipulated (including images) to support our conclusions. No data, text, or theories by others are presented as if they were our own. The submission has been received explicitly from all co‐authors. And authors whose names appear on the submission have contributed sufficiently to the scientific work and therefore share collective responsibility and accountability for the results. This article does not contain any studies with human participants or animals performed by any of the authors. Informed consent was obtained from all individual participants included in the study.

## CONFLICT OF INTEREST

The authors declare that they have no conflict of interest.
